# Sequential disruption of ALV host receptor genes reveals no sharing of receptors between ALV subgroups A, B, and J

**DOI:** 10.1186/s40104-019-0333-x

**Published:** 2019-04-02

**Authors:** Hong Jo Lee, Kyung Je Park, Kyung Youn Lee, Yongxiu Yao, Venugopal Nair, Jae Yong Han

**Affiliations:** 1 Department of Agricultural Biotechnology, College of Agriculture and Life Sciences, and Research Institute of Agriculture and Life Sciences, Seoul National University, Seoul, 08826 Korea; 2 The Pirbright Institute, Woking, Surrey GU24 0NF UK

**Keywords:** Avian leukosis virus, CRISPR/Cas9, Genome editing, Host receptor, TVA

## Abstract

**Background:**

Previously, we showed that targeted disruption of viral receptor genes in avian leukosis virus (ALV) subgroups using clustered regularly interspaced short palindromic repeats (CRISPR)/CRISPR-associated protein 9 (Cas9))-based genome editing confers resistance to ALV subgroups B and J. Here, we used the same strategy to target the receptor expressed by ALV subgroup A (TVA) and generate chicken cells resistant to infection by this virus.

**Results:**

CRISPR/Cas9-based disruption of exon 2 within the *tva* gene of DF-1 fibroblasts conferred resistance to infection by ALV subgroup A regardless of whether frameshift mutations were introduced during editing. Conversely, overexpression of the wild-type TVA receptor (wtTVA) by *tva*-modified DF-1 clones restored susceptibility to ALV subgroup A. The results confirm that exon 2, which contains the low-density lipoprotein receptor class A domain of TVA, is critical for virus entry. Furthermore, we sequentially modified DF-1 cells by editing the *tva*, *tvb,* and Na^+^/H^+^ exchange 1 (*chNHE1*) genes, which are the specific receptors for ALV subgroups A, B, and J, respectively.

**Conclusions:**

Simultaneous editing of multiple receptors to block infection by different subgroups of ALV confirmed that ALV subgroups A, B, and J do not share host receptors. This strategy could be used to generate cells resistant to multiple viral pathogens that use distinct receptors for cell entry.

**Electronic supplementary material:**

The online version of this article (10.1186/s40104-019-0333-x) contains supplementary material, which is available to authorized users.

## Introduction

Avian leukosis viruses (ALV) are pathogenic avian retroviruses that cause major neoplastic diseases in many poultry flocks, resulting in significant economic losses to the global poultry industry [1]. While a number of poultry breeding companies have been successful at eradicated ALV infections from flocks, continued spread of ALV in some Asian countries remains an issue for the poultry market [2–5].

ALVs are divided into different subgroups based primarily on the sequence of the envelop glycoprotein, which is the major determinant of the receptor interactions required for virus entry, host range, and virus neutralization [6]. Each ALV subgroup targets specific host cell receptors to interact with the envelop glycoproteins; thus, viral entry into cells depends largely on dominant expression of a particular host receptor. ALV subgroup A enters host cells via the TVA receptor, which is a low-density lipoprotein receptor (LDLR) [7]. ALV subgroups B, D, and E enter cells via the TVB receptor, a tumor necrosis factor receptor (TNFR)-related protein [8–10]. ALV subgroup C uses the TVC receptor, which is related to the mammalian butyrophilins [11], and ALV subgroup J uses chicken Na^+^/H^+^ exchanger type 1 (chNHE1) [12].

Genetic variations in host receptor genes determine the susceptibility of chickens to infection by different ALV subgroups. A single base pair mutation resulting in a cysteine to tryptophan mutation or a four base pair insertion into exon 1 of the *tva* gene confers resistence to ALV subgroup A viruses [13]. In addition, intronic deletions within the *tva* receptor gene that disrupt mRNA splicing confer resistance to ALV subgroup A viruses [14, 15]. In addition, a naturally occurring premature stop codon or a single amino acid substitution within the *tvb* gene leads to a marked reduction in susceptibility to ALV subgroups B, D, and E [16, 17]. Furthermore, a single nucleotide substitution resulting in premature stop codon in the *tvc* allele confers resistance to ALV subgroup C [11]. Finally, the tryptophan residue at position 38 (Trp38) of chNHE1 is critical for cell entry by ALV subgroup J [18].

Genetic analyses by our group led to development of cell lines that are resistant to infection by ALV subgroups B and J [19, 20]. Here, we used the same CRISPR/Cas9-based editing approach to develop a new cell line that is resistant to infection by ALV subgroup A. Furthermore, we applied the technology to perform sequential disruption of the *tva*, *tvb,* and chNHE1 receptors to develop a cell line that is resistant to all three ALV subgroups.

## Materials and methods

### Construction of CRISPR/Cas9 expression vectors

**Fig. 1:**
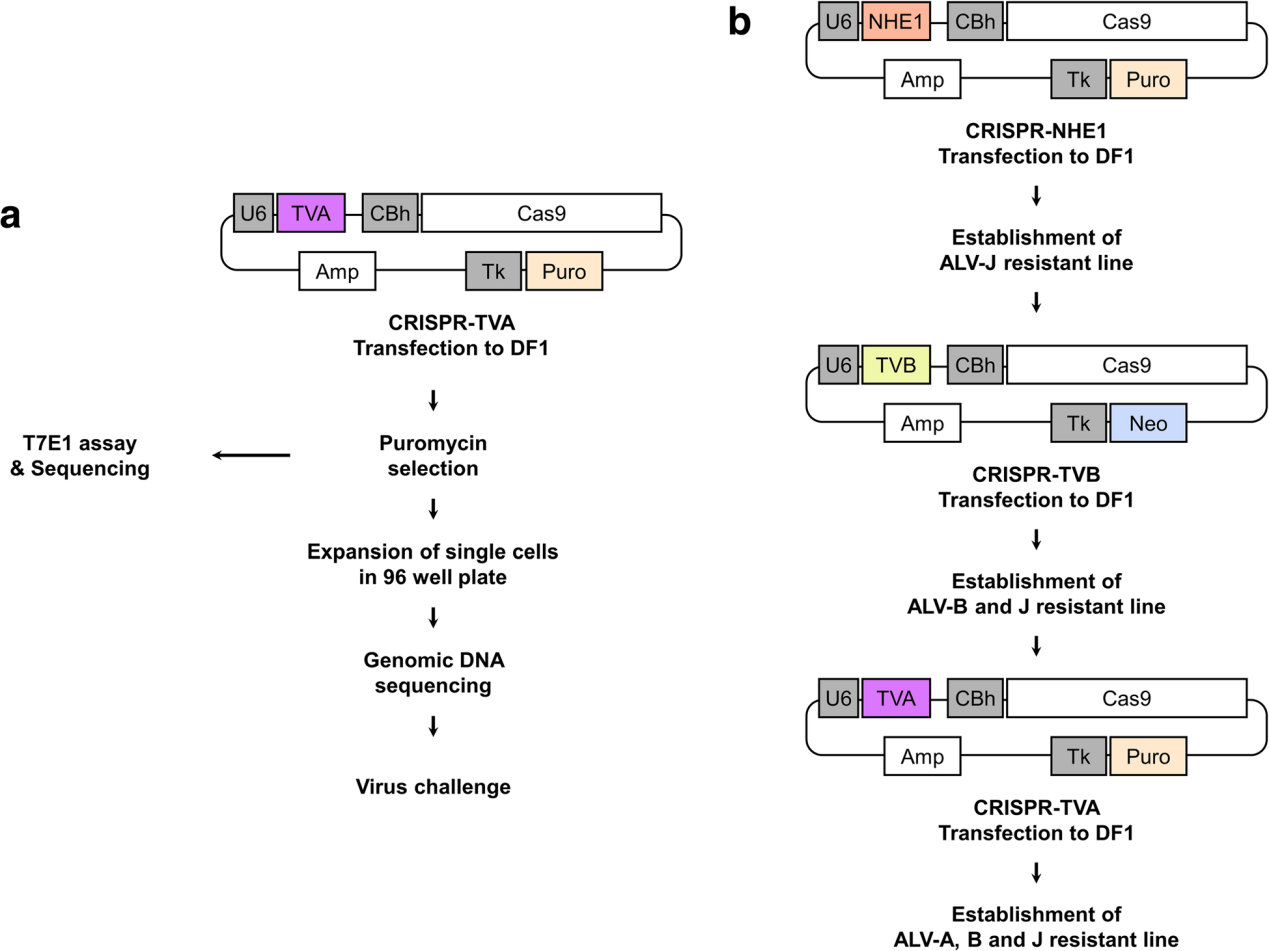
Schematic representation showing genome editing of Avian Leukosis Virus (ALV) host receptors. **a** DF-1 cells were transfected with CRISPR/Cas9 vectors containing Cas9 protein-coding sequences, *tva*-targeting guide RNA, and puromycin resistance genes. After puromycin selection a T7E1 assay and sequence analysis were performed. In addition, single *tva*-modified DF-1 cells were cultured the *tva* gene sequenced. Clones were then infected with ALV subgroup A produced by RCAS-A-GFP vector-transfected DF-1 cells. **b** Schematic representation showing sequential disruption of host receptors for ALV subgroups J, B, and A. TVA, *tva* targeting gRNA; TVB, *tvb* targeting gRNA; NHE1, *chNHE1* targeting gRNA; U6, human U6 promoter; CBh, chicken beta-actin short promoter; Amp, ampicillin; Tk, thymidine kinase promoter; Neo, neomycin resistance gene; Puro, puromycin resistance gene

We constructed all-in-one CRISPR/Cas9 vectors targeting *tva*, with minor modifications. The CRISPR kit used for constructing multiplex CRISPR/Cas9 vectors was a gift from Takashi Yamamoto (Addgene Kit #1000000054) [21]. A puromycin resistance gene under the regulation of a thymidine kinase promoter were inserted into CRISPR/Cas9 vectors by *NotI* digestion and ligation (Fig. 1a) (New England Biolabs, Ipswich, MA, USA). To insert guide RNA sequences into CRISPR/Cas9 vectors, we synthesized sense and antisense oligonucleotides (Bionics, Seoul, Korea) and carried out annealing using the following thermocycling conditions: 30 s at 95 °C, 2 min at 72 °C, 2 min at 37 °C, and 2 min at 25 °C. The oligonucleotides used are listed in Table 1.

**Table 1:** Oligos used in the study

Oligo ID	Sequence (5′→3′)
TVA#1 F	CACCGGCGACGATGGACGGGACGAG
TVA#1 R	AAACCTCGTCCCGTCCATCGTCGCC
TVA#4 F	CACCGCGCTGGAGTGGCTCTGCGAC
TVA#4 R	AAACGTCGCAGAGCCACTCCAGCGC
TVA seq F	ACACTGACAGCGAGGCGTGC
TVA seq R	ACCTCTCCGCACGACGTTCT
wtTVA F	AAGCTTCCGGCGGGCCCGGGGCCGGCATG
wtTVA R	GCGGCCGCAGGCAAAAAGAGTGAGGGAATTCC
mutaTVA(+ 1) F	CTGCGACGATGGACGGGACCGAGTGGGGCTGCGGAGCGAG
mutaTVA(+ 1) R	CTCGCTCCGCAGCCCCACTCGGTCCCGTCCATCGTCGCAG
mutaTVA(−3) F	CGACTGCGACGATGGACGGGAGTGGGGCTGCGGAGCGAGC
mutaTVA(−3) R	GCTCGCTCCGCAGCCCCACTCCCGTCCATCGTCGCAGTCG
mutaTVA(∆1–6) F	ATCCCGACTGCGACGATGGAAAGTGGGGCTGCGGAGCGAG
mutaTVA(∆1–6) R	CTCGCTCCGCAGCCCCACTTTCCATCGTCGCAGTCGGGAT

### Culture of DF-1 chicken fibroblasts

DF-1 cells were maintained and subpassaged in Dulbecco’s minimum essential medium (DMEM; Hyclone, Logan, UT, USA), supplemented with 10% fetal bovine serum (FBS; Hyclone) and 1× antibiotic–antimycotic (ABAM; Thermo Fisher–Invitrogen, Carlsbad, CA, USA). DF-1 cells were cultured in an incubator at 37 °C in an atmosphere of 5% CO_2_ at 60%–70% relative humidity.

### Transfection and drug selection of DF-1 cells

CRISPR/Cas9 vectors (3 μg) were mixed with Lipofectamine 2000 reagent (Thermo Fisher–Invitrogen) in Opti-MEM (Thermo Fisher–Invitrogen), and the mixture was applied to 5 × 10^5^ DF-1 cells. Then, 6 h after transfection, transfection mixtures were replaced with DF-1 culture medium. G418 (300 μg/mL) and puromycin (1 μg/mL) were added to the culture medium 1 d after transfection. The complete selection period required up to 7 d.

### T7E1 assay

Genomic DNA was extracted from DF-1 cells after puromycin selection. Genomic regions encompassing the CRISPR/Cas9 target sites were amplified using specific primer sets. PCR analysis of the targeted loci was examined in a total volume of 20 μL containing 100 ng genomic DNA, 10× PCR buffer (BioFACT, Daejeon, Korea), 0.4 μL dNTPs (10 mmol/L each), 10 pmol of each primer, and 0.5 U Taq polymerase (BioFACT) under the following thermocycling conditions: 2 min at 95 °C, followed by 35 cycles of 20 s at 95 °C, 40 s at 65 °C, and 30 s at 72 °C, and a final 5 min at 72 °C. Primers are listed in Table 1. The PCR amplicons were re-annealed to form a heteroduplex DNA structure after denaturation. Subsequently, the heteroduplex amplicons were treated with 5 units T7E1 endonuclease (New England Biolabs) for 20 min at 37 °C and then analyzed by 1% agarose gel electrophoresis.

### Culture of single DF-1 cells and genomic DNA sequencing

After drug selection, the heterozygous DF-1 cells transfected with each CRISPR/Cas9 vectors were diluted with DF-1 culture medium concentration at one cell per 100 μL, then single DF-1 cells were seeded in individual wells of a 96-well plate. We checked the wells each day after seeding, and when the cells in each well reached confluency, subpassaged the cells into a 48-well plate. These cells were then used for genomic DNA extraction. The genomic regions encompassing the target sites in DF-1 were amplified using specific primer sets (Table 1). PCR analysis of the targeted loci was examined in same condition with T7E1 analysis. And the PCR products were sequenced using the ABI Prism 3730 XL DNA Analyzer (Thermo Fisher–Applied Biosystems, Foster City, CA, USA). The sequences were compared against assembled genomes using NCBI BLAST.

### Construction of tva receptor overexpression vector

The wild type TVA coding sequence was cloned from DF-1 cDNA using wtTVA forward and reverse primers (Table 1), and cloned into *piggyBac*-CMV-GFP-FRT vector [22] by *HindIII* and *NotI* digestion and ligation. The sequences were compared against assembled genomes using NCBI BLAST.

### Sequential disruptions of ALV host receptors and genomic DNA sequencing

Trp38 of *chNHE1*-modified DF-1 clone (N3Pss#12) [20] was transfected with the CRISPR/Cas9 vector targeting *tvb* gene (TVB#2) [19]. And the DF-1 was seeded in individual wells of a 96-well plate with 100 μL DF-1 culture medium. After confirmation of mutations of *tvb* targeting region in the DF-1 clones by sequencing analysis, the CRISPR/Cas9 vectors targeting *tva* gene were transfected into the *chNHE1* and *tvb*-modified DF-1 clones subsequently and individual DF-1 clones were cultured. And targeting region of *tva* gene of the DF-1 clones was analyzed by sequencing analysis (Fig. 1b).

### Virus production and infection

RCAS-A-EGFP DNA, RCAS-B-EGFP DNA and RCAS-J-EGFP DNA (5 μg each) was mixed with Lipofectamine 2000 reagent (Thermo Fisher–Invitrogen) in Opti-MEM (Thermo Fisher–Invitrogen) in separate tubes, and the mixture was applied to 1 × 10^6^ DF-1 cells each. The mixture was replaced with DF-1 culture medium 6 h after transfection. One day after transfection, we could observe green fluorescence in DF-1 cells, indicating virus production. Cells were passaged, and the medium was changed 1 d after passaging. One day later, the medium containing viruses was harvested and frozen at − 70 °C until further use. For viral infection, the medium containing viruses was thawed to 37 °C and added to the cultured individual DF-1 clones. Four days post-infection (dpi), DF-1 clones were checked under fluorescence microscopy (TU-80; Nikon, Tokyo, Japan) and analyzed using FACS Calibur software (BD Biosciences, San Jose, CA, USA).

### Amino acid sequence analysis

The amino acid sequences of quail Tva receptor with four chicken Tva isoforms were aligned using the UniProt consortium.

### Site directed mutagenesis PCR

cDNA encoding full length of TVA receptor cloned in pGEM T easy vector (Promega) is used as a template. Mutations were introduced by site-directed mutagenesis PCR, a total volume of 100 μL containing 10 ng template vector, 10× PCR buffer (enzynomics), 0.4 μL dNTPs (10 mmol/L each), 10 pmol of each primer, and 2.5 U Pfu polymerase (enzynomics) under the following thermocycling conditions: 3 min at 95 °C, followed by 35 cycles of 30 s at 95 °C, 30 s at 50 °C, and 4 min at 72 °C, and a final 15 min at 72 °C. 3 types of primer sets are used and mutagenic oligonucleotides used are shown in Table 1. The amplicons were treated with 10 U of *DpnI* enzyme (New England Biolabs) for 1 h at 37 °C and then purified with Wizard SV Gel and PCR Clean-up system (Promega). For phosphorylation, 2 μL buffer A (Thermofisher), 10 U T4 polynucleotide kinase (Thermofisher), 2 μL 10 mmol/L ATP (Thermofisher) and 14 μL of purified DNA for 1 h at 37 °C. The phosphorylated PCR products were self-ligated using T4 ligase (Takara), and the mutated TVA sequences were analyzed using the ABI Prism 3730 XL DNA Analyzer (Thermo Fisher–Applied Biosystems, Foster City, CA, USA). Then, the three mutated TVA coding sequences were cloned into *piggyBac*-CMV-GFP-FRT vector by *HindIII* and *NotI* digestion and ligation. The sequences were compared against assembled genomes using NCBI BLAST (http://blast.ncbi.nlm.nih.gov).

### Statistical analyses

Statistical Analysis System (SAS) software (SAS Institute, Cary, NC, USA) was used for the analysis of ALV subgroup A, B and J susceptibility. Treatments were compared using the least-squares method or Duncan’s method, and the significance of the main effects was determined using analysis of variance (ANOVA) in the SAS package. A *P*-value < 0.05 indicated a statistically significant difference.

## Results

### Targeted mutation of the *tva* gene in chicken DF-1 fibroblasts

**Fig. 2:**
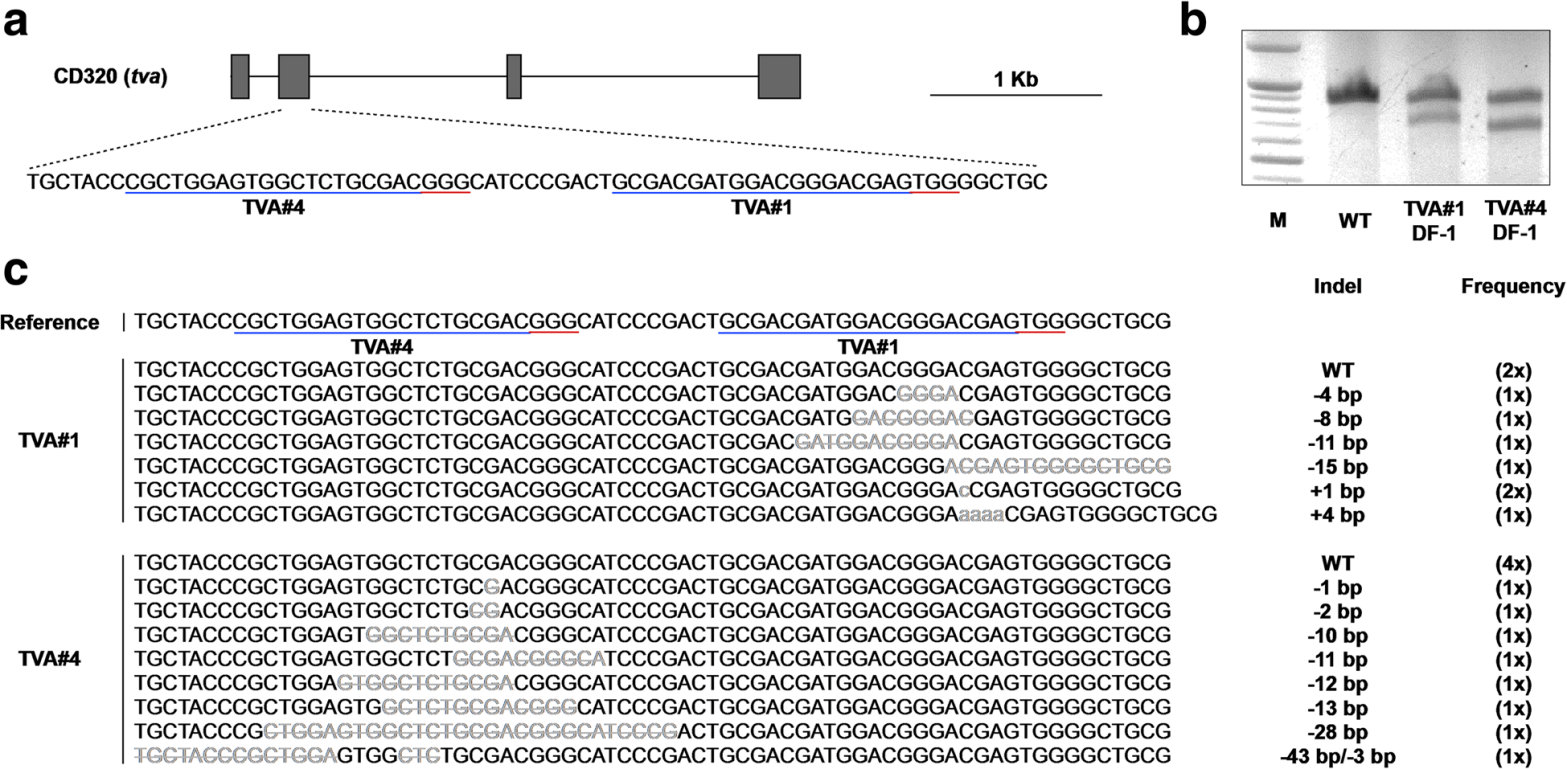
Targeted disruption of the *tva* gene in DF-1 fibroblasts using the CRISPR/Cas9 system. **a** The gene structure of *tva* (CD320) and the locus targeted by CRISPR/Cas9 vectors (TVA#1 and TVA#4). Scale bar = 1 kb. **b** T7E1 assay of DF-1 cells transfected with the TVA#1 and TVA#4 CRISPR/Cas9 vectors. Wild type (WT) DF-1 fibroblasts were used as the control. **c** Sequence analysis of transfected DF-1 cells using the TA cloning method. Gray lowercase letters indicate insertions, and gray letters with lines indicate deletions. Indel mutations and their frequencies are presented. Blue bars indicate guide RNA binding sites and red bars indicate protospacer adjacent motif (PAM) sequences

To examine whether disrupting the TVA receptor confers resistance to ALV subgroup A, we used the CRISPR/Cas9 system to introduce a targeted mutation in the *tva* gene of chicken DF-1 fibroblasts. First, we constructed two CRISPR/Cas9 vectors (TVA#1 and TVA#4) targeting exon 2 of the *tva* gene to introduce frameshift mutations into the TVA receptor (Fig. 2a). The CRISPR/Cas9 vectors were then introduced into DF-1 fibroblasts and transfected cells were selected for drug resistance. The results of a T7E1 assay showed that cleaved bands were detected only in DF-1 fibroblasts transfected with each of the CRISPR/Cas9 vectors, indicating that the CRISPR/Cas9 vectors efficiently induced nucleic acid mutations at the targeted locus of the *tva* gene (Fig. 2b). Sequence analysis of the targeted locus [10] using the TA cloning method confirmed that the DF-1 fibroblasts harbored indel mutations at the targeted regions with 77.8% (7/9) and 66.7% (8/12) efficiency, respectively (Fig. 2c).

### Establishment of *tva*-modified DF-1 clones and subsequent challenge with ALV subgroup A

To examine susceptibility of *tva*-modified DF-1 fibroblasts to infection by ALV subgroup A, we generated single clones from DF-1 fibroblasts transfected with TVA#1 and TVA#4. We generated eight clones from the two experimental groups, and sequence analysis of the PCR products derived from each clone showed that six of them harbored nucleotide mutations in the *tva* gene (Additional file 1: Figure S1). Three of these (TVA#1–6, TVA#1–8, and TVA#4–4) harbored frameshift mutations (including deleted- or inserted amino acids) at the targeted locus that generated a premature stop codon in the *tva* gene. Three other clones (TVA#1–2, TVA#1–5, and TVA#1–7) harbored deletion-, insertion- or substitution- at the targeted locus that did not introduce a frameshift, while two others (TVA#4–2 and TVA#4–9) harbored the wild-type (WT) *tva* allele (Table 2).

**Table 2:** Sequences of *tva*-modified DF-1 clones

ID	Sequence (5′→3′)	Indel
WT	TGCTACCCGCTGGAGTGGCTCTGCGACGGGCATCCCGACTGCGACGATGGACGGGACGAGTGGGGCTGCG	WT
TVA#1–2		1 modif/ − 6 bp
TVA#1–5		−3 bp
TVA#1–6	TGCTACCCGCTGGAGTGGCTCTGCGACGGGCATCCCGACTGCGACGATGGACGGGAcCGAGTGGGGCTGCG	+ 1 bp
TVA#1–7		−42 bp
TVA#1–8		−16 bp
TVA#4–2	TGCTACCCGCTGGAGTGGCTCTGCGACGGGCATCCCGACTGCGACGATGGACGGGACGAGTGGGGCTGCG	WT
TVA#4–4		−13 bp
TVA#4–9	TGCTACCCGCTGGAGTGGCTCTGCGACGGGCATCCCGACTGCGACGATGGACGGGACGAGTGGGGCTGCG	WT

Underlining indicates guide RNA recognition sites and protospacer-adjacent motif sequences

Strikethroughs indicate deleted nucleotides and the lowercase letter indicates an inserted nucleotide. The italicized bold letter indicates a modified mutation

**Fig. 3:**
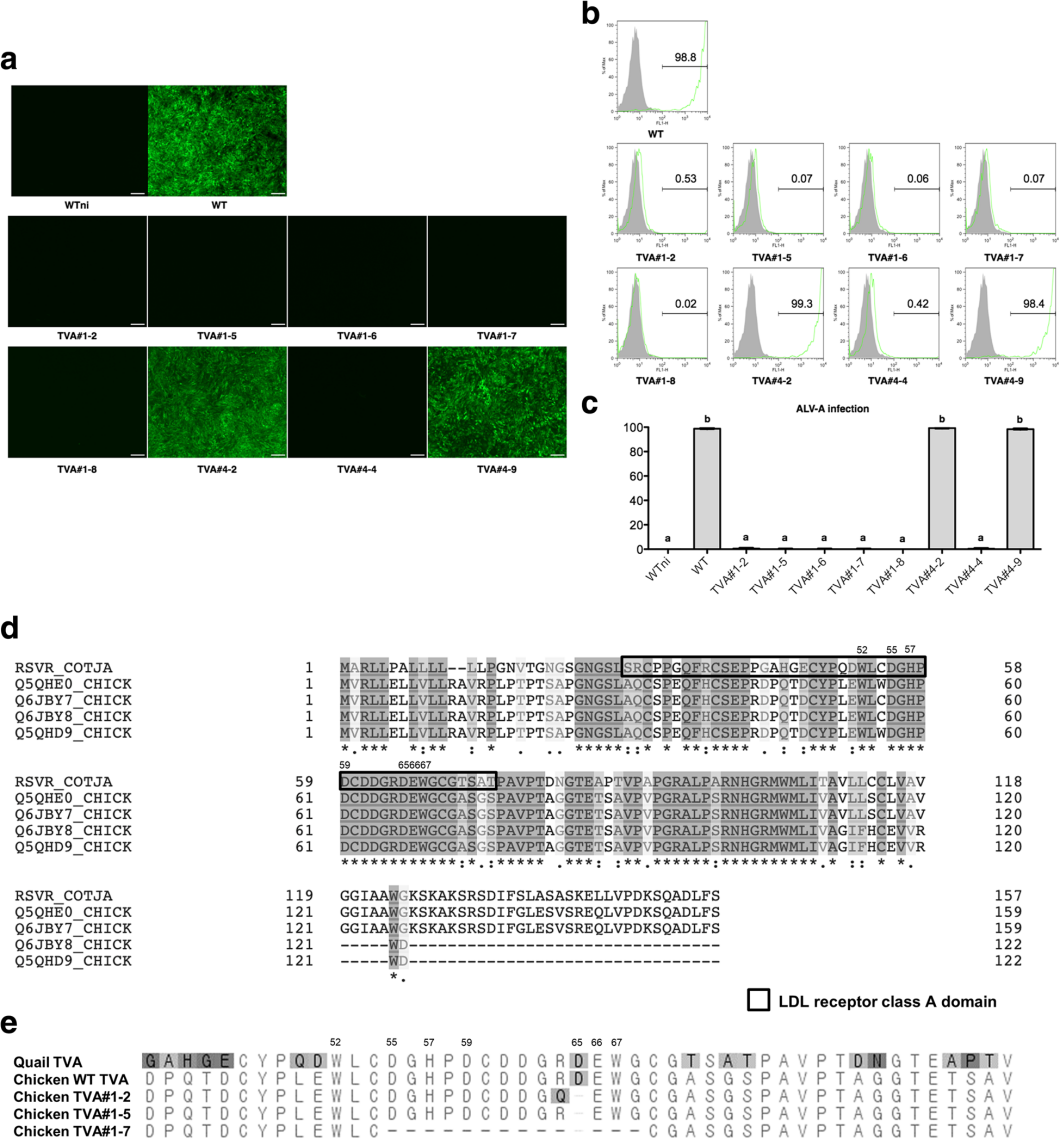
Infection of *tva*-modified DF-1 clones with avian leukosis virus subgroup A, followed by flow cytometry analysis. **a** Expression of GFP by virus-infected DF-1 clones. Eight DF-1 clones were examined under a fluorescence microscope. Scale bar = 100 μm. **b**, **c** Flow cytometry analysis of virus-infected DF-1 clones. Gray peaks indicate the population of wild-type (WT) cells not infected with ALV subgroup A (negative control) and green peaks indicate the population of ALV subgroup A-infected DF-1 clones. The numbers on the histograms represent the mean percentage of cells within that population of triplicate replications. WT DF-1 fibroblasts were used as the control (positive control). **c** Data represent the mean ± SEM of triplicate replications. Different letters (a, b) indicate significant differences (*P* < 0.05). **d** Multiple-sequence alignment of the four chicken Tva receptor isoforms with the quail Tva receptor. Conserved residues are indicated by the asterisks (identical residues) and dots (similar residues) below the aligned sequences. The quadrangle denotes the viral receptor domain of quail Tva, and the numbers on LDLR class A domain denote the order of quail Tva amino acid sequences. Sequence alignment analysis was conducted using the UniProt consortium. **e** Comparative analysis of deduced amino acids of *tva*-modified DF-1 clones. Quail Tva and chicken Tva amino acid are used as references. Tva amino acids of the in-frame *tva*-modified DF-1 clones are presented. TVA#1–2 clone has mutations on the 64^th^ and the 65^th^ positions of Tva amino acid, TVA#1–5 clone has mutation on 65^th^ position of Tva amino acid and TVA#1–7 clone has large deletions on from the 55^th^ to 68^th^ positions of Tva amino acid sequences. Different amino acids are highlighted. The numbers denote the order of quail Tva amino acid sequences

Next, we infected each DF-1 clone with ALV subgroup A to examine resistance. Based on expression of green fluorescent protein (GFP) by the marker virus, we found that DF-1 clones harboring nucleic acid mutations in the *tva* gene (TVA#1–2, TVA#1–5, TVA#1–6, TVA#1–7, TVA #1–8, and TVA#4–4) did not express GFP, suggesting that ALV subgroup A did not enter these cells; by contrast, virus did enter WT DF-1 control cells (Fig. 3a). Subsequent flow cytometry analysis confirmed that expression of GFP was significantly higher in DF-1 clones harboring the WT *tva* gene (TVA#4–2, and TVA#4–9), indicating that genetic modification of the *tva* gene confers resistance to ALV subgroup A, regardless of the presence of frameshift mutations (Fig. 3b and c). Comparative analysis showed that quail Tva and chicken Tva are highly conserved particularly in the LDLR class A domain (LDLa) (Fig. 3d), and the TVA#1–2 clone has mutations on the 64^th^ and 65^th^ positions of Tva amino acid, TVA#1–5 clone has mutation on the 65^th^ position of Tva amino acid and TVA#1–7 clone has large deletions on from 55th to 68th positions of Tva amino acid sequences (Fig. 3e).

### Overexpression of the WT TVA receptor by *tva*-modified DF-1 clones restores susceptibility to infection by ALV subgroup A

**Fig. 4:**
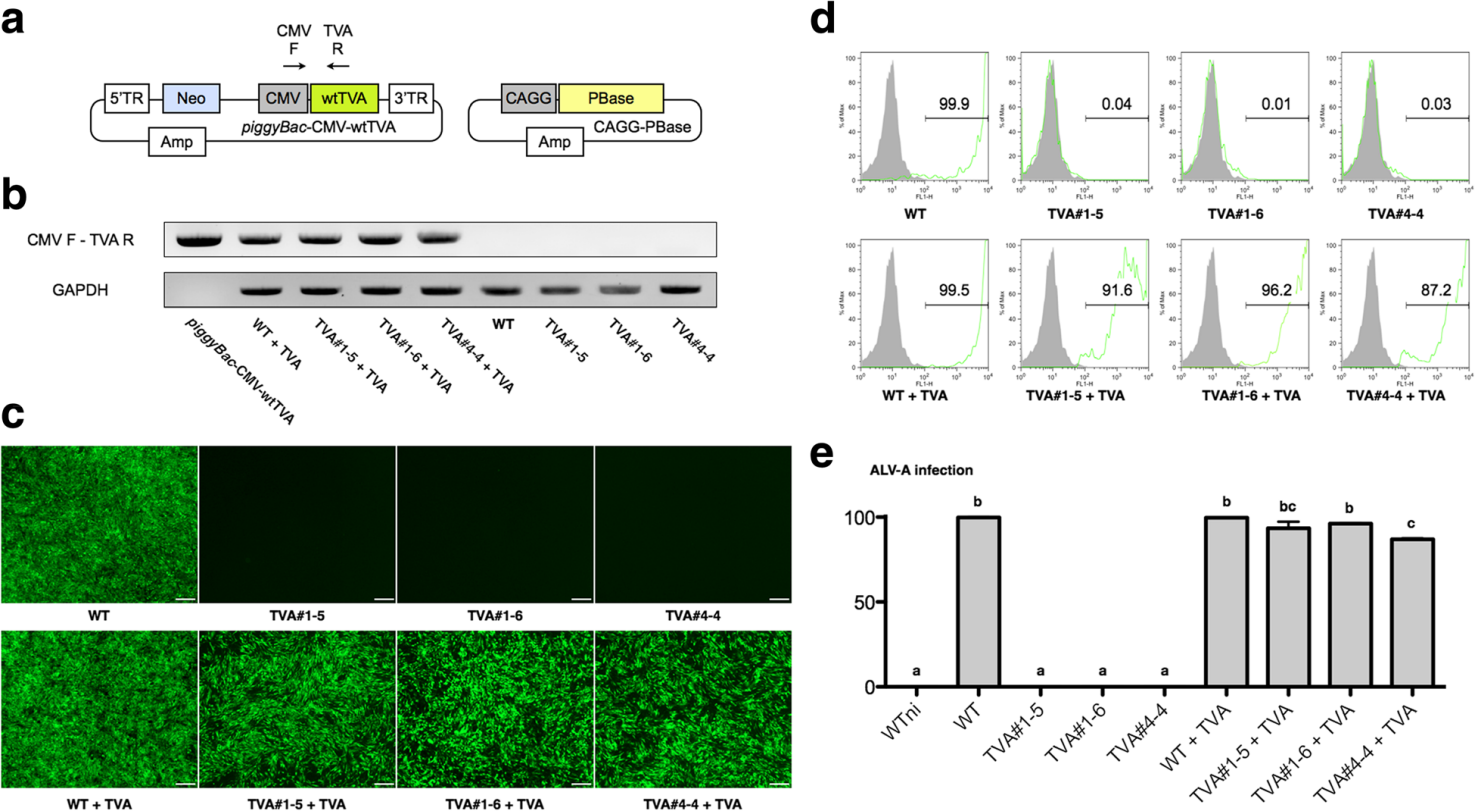
Overexpression of the wild-type (WT) TVA receptor restores susceptibility of *tva*-modified DF-1 clones to infection by ALV subgroup A. **a** Schematic representation showing the wild-type TVA receptor (WT TVA) overexpression cassette. 5′-TR: *piggyBac* 5′-terminal repeat; Neo: neomycin resistant gene; CMV: cytomegalovirus promoter; WT TVA: WT TVA coding sequence; 3′-TR: *piggyBac* 3′-terminal repeat; Amp: ampicillin resistant gene; CAGG: cytomegalovirus (CMV) enhancer fused to the chicken beta-actin promoter; PBase: *piggyBac* transposase coding sequence. **b** Analysis of genomic DNA to confirm integration of the WT TVA overexpression cassette under specific PCR conditions. GAPDH was used as a control. **c** Expression of GFP by virus-infected DF-1 clones. Three DF-1 clones (TVA#1–5, TVA#1–6, and TVA#4–4) and WT DF-1 cells were transfected with the WT TVA overexpression cassette and then infected with ALV subgroup A. GFP expression by each group was examined under a fluorescence microscope. Scale bar = 100 μm. **d**, **e** Flow cytometry analysis of virus-infected DF-1 clones. Gray peaks indicate the population of WT cells not infected with ALV subgroup A (negative control) and green peaks indicate the population of DF-1 clones infected with ALV subgroup A. The numbers on the histograms represent the mean percentage of cells within that population of triplicate replications. WT DF-1 fibroblasts were used as the control (positive control). Different letters (a, b and c) indicate significant differences (*P* < 0.05)

Next, we examined whether overexpressing WT TVA restores susceptibility of *tva*-modified DF-1 clones to infection by ALV subgroup A. For this, we constructed a *piggyBac* transposon-based WT TVA overexpression vector (Fig. 4a). Next, we integrated a WT TVA overexpression cassette into *tva*-modified DF-1 clones (TVA#1–5, TVA#1–6, and TVA#4–4) and WT DF-1 using *piggyBac* transposase. Analysis of genomic DNA revealed that WT TVA vector-transfected DF-1 lines contained the WT TVA overexpression cassette (Fig. 4b).

Next, infected DF-1 cell lines with ALV subgroup A and examined their susceptibility to infection. GFP expression was detected in WT TVA-overexpressing cell lines (TVA#1–5 + TVA, TVA#1–6 + TVA, and TVA#4–4 + TVA) (Fig. 4c). Quantitative analysis using flow cytometry showed that GFP expression by WT TVA-overexpressing groups was significantly higher than that by *tva*-modified DF-1 clones, although there were differences between the WT TVA-overexpressing groups (Fig. 4d and e).

**Fig. 5:**
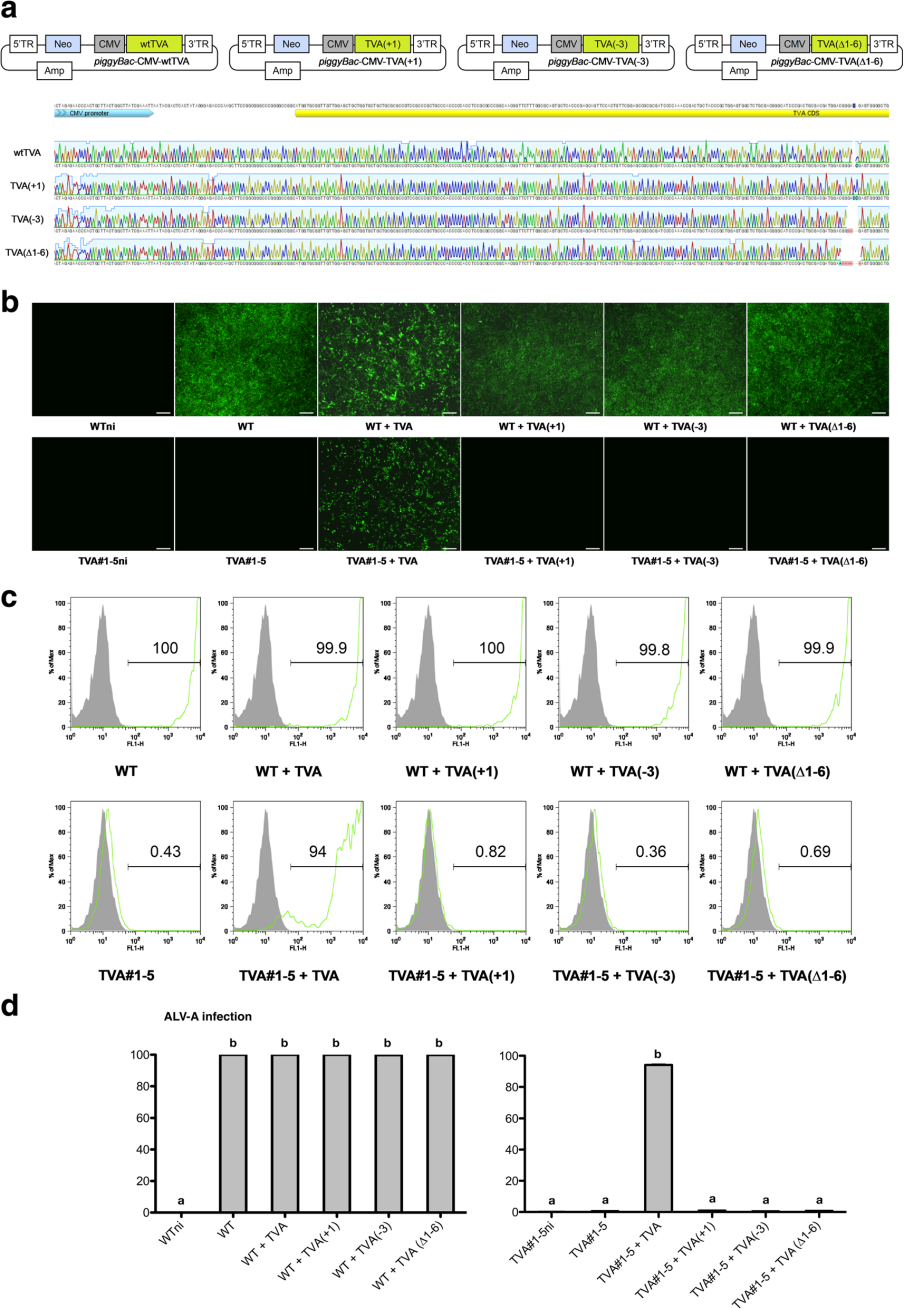
**a** Schematic representation showing the wild-type TVA receptor (WT TVA) and modified TVA receptor (TVA(+ 1), TVA(− 3) and TVA(∆1–6)) overexpression cassette and sequencing results of the constructed vector. CMV promoter and TVA CDS sequences are described, and modified nucleic acids are highlighted. 5′-TR: *piggyBac* 5′-terminal repeat; Neo: neomycin resistant gene; CMV: cytomegalovirus promoter; 3′-TR: *piggyBac* 3′-terminal repeat; Amp: ampicillin resistant gene; CAGG: cytomegalovirus (CMV) enhancer fused to the chicken beta-actin promoter; PBase: *piggyBac* transposase coding sequence. **b** Expression of GFP by virus-infected DF-1 clones. WT TVA or modified TVA receptors overexpessing WT DF-1 cell lines (WT, WT + TVA, WT + TVA(+ 1), WT + TVA(− 3) and WT + TVA(∆1–6)) and *tva*-modified DF-1 clones (3 bp deletion on *tvb*) (TVA#1–5, TVA#1–5 + TVA, TVA#1–5 + TVA (+ 1), TVA#1–5 + TVA(− 3) and TVA#1–5 + TVA(∆1–6)) were examined under a fluorescence microscope. Scale bar = 100 μm. **c**, **d** Flow cytometry analysis of virus-infected DF-1 clones. Gray peaks indicate the population of the DF-1 not infected with ALV subgroup A (negative control; WTni and TVA#1-5ni) and green peaks indicate the population of ALV subgroup A-infected DF-1 clones. The numbers on the histograms represent the mean percentage of cells within that population of triplicate replications. WT DF-1 fibroblasts were used as the control (positive control). **d** Data represent the mean ± SEM of triplicate replications. Different letters (a, b) indicate significant differences (*P* < 0.05)

To further confirm the consequence of mutations on *tva* for ALV subgroup A susceptibility, we establihsed the DF-1 clones that express modified TVA receptor by *piggyBac* transposon (Fig. 5a), then examined their susceptibility to infection. As results, GFP expression was not significantly reduced in the modified TVA-overexpressing WT DF-1 cell lines (WT + TVA (+ 1), WT + TVA (− 3), WT + TVA (∆1–6)). And, we established the modified TVA receptor-overexpressing DF-1 cell lines from the DF-1 clones that already have a 3-bp pair deletion on *tva* gene (TVA#1–5). The results of viral challenge in the cell lines showed that ALV subgroup A susceptibility can not be restored by overexpression of the modified TVA receptors in *tva*-modified DF-1 clone (TVA#1–5 + TVA (+ 1), TVA#1–5 + TVA (− 3), TVA#1–5 + TVA (∆1–6)) (Fig. 5b, c and d).

### Sequential disruption of host receptors for ALV subgroups A, B and J and subsequent virus challenge

In previous studies, we showed that artificially generating a premature stop codon in the cysteine-rich domain (CRD) of the *tvb* gene confers resistance to ALV subgroup B [19], and that deletion of *chNHE1* Trp38 confers resistance to ALV subgroup J [20]. Therefore, we examined whether sequential editing of the *tva*, *tvb,* and *chNHE1* genes confers resistance to ALV subgroups A, B, and J simultaneously.

To induce sequential disruption of host receptors (*tva*, *tvb*, and *chNHE1*), we used a N3Pss#12 DF-1 clone in which *chNHE1* Trp38 was deleted [20]. Sequential transfection of CRISPR/Cas9 vectors targeting the *tvb* and *tva* genes into the *chNHE1*-modified clone generated DF-1 clones harboring nucleic acid mutations in the *tva*, *tvb,* and *chNHE1* genes. First, we confirmed that the N3Pss#12 DF-1 clone harbored a mutation in *chNHE1* by sequencing a PCR product amplified from the target region (Additional file 1: Figure S2A) [20]. Next, we confirmed that the N3Pss#12 + TVB#2 clone harbored an 8-bp deletion in the *tvb* gene that generated a premature stop codon (Additional file 1: Figure S2B). Finally, we confirmed that the three DF-1 clones (N3Pss#12 + TVB#2 + TVA#1–6, N3Pss#12 + TVB#2 + TVA#1–8, and N3Pss#12 + TVB#2 + TVA#4–8) established by sequential transfection of CRISPR/Cas9 vectors targeting *tvb* and *tva* harbored the same mutations in *tvb* as the N3Pss#12 + TVB#2 clones, as well as the same deletion mutations in the *tva* gene (Additional file 1: Figure S2C) and the mutations in the *chNHE1* (Table 3).

**Table 3:** Sequences of *chNHE1*, *tvb*, and *tva*-modified DF-1 clones

ID	Sequence (5′→3′)	Indel
WT		
*chNHE1*	CCGACGCCACGCGTGTCTCCGAGCCCACCTGGGAGCAGCCGTGGGGAGAGCCCGGGGGTATCACCGCC	WT
*tvb*	AAAGACGAGTACACCGAGTATCCAAATGACTTTCCCAAGTGCCTGGGCTGCCGGACGTGTAGGGAAGGTAT	WT
*tva*	TGCTACCCGCTGGAGTGGCTCTGCGACGGGCATCCCGACTGCGACGATGGACGGGACGAGTGGGGCTGCG	WT
N3Pss#12		
*chNHE1*		−3 bp, T96G
*tvb*	AAAGACGAGTACACCGAGTATCCAAATGACTTTCCCAAGTGCCTGGGCTGCCGGACGTGTAGGGAAGGTAT	WT
*tva*	TGCTACCCGCTGGAGTGGCTCTGCGACGGGCATCCCGACTGCGACGATGGACGGGACGAGTGGGGCTGCG	WT
N3Pss#12 + TVB#2		
*chNHE1*		−3 bp, T96G
*tvb*		−8 bp
*tva*	TGCTACCCGCTGGAGTGGCTCTGCGACGGGCATCCCGACTGCGACGATGGACGGGACGAGTGGGGCTGCG	WT
N3Pss#12 + TVB#2 + TVA#1–6		
*chNHE1*		−3 bp, T96G
*tvb*		−8 bp
*tva*		−46 bp
N3Pss#12 + TVB#2 + TVA#1–8		
*chNHE1*		−3 bp, T96G
*tvb*		−8 bp
*tva*		−4 bp
N3Pss#12 + TVB#2 + TVA#4–8		
*chNHE1*		−3 bp, T96G
*tvb*		−8 bp
*tva*		−6 bp

Underling indicates guide RNA recognition sites and protospacer-adjacent motif sequences

Strikethroughs indicate deleted nucleotides and italicized bold letters indicate modified mutations

### Challenge of *chNHE1*, *tvb*, and *tva*-modified DF-1 clones with ALV subgroup A, B and J

**Fig. 6:**
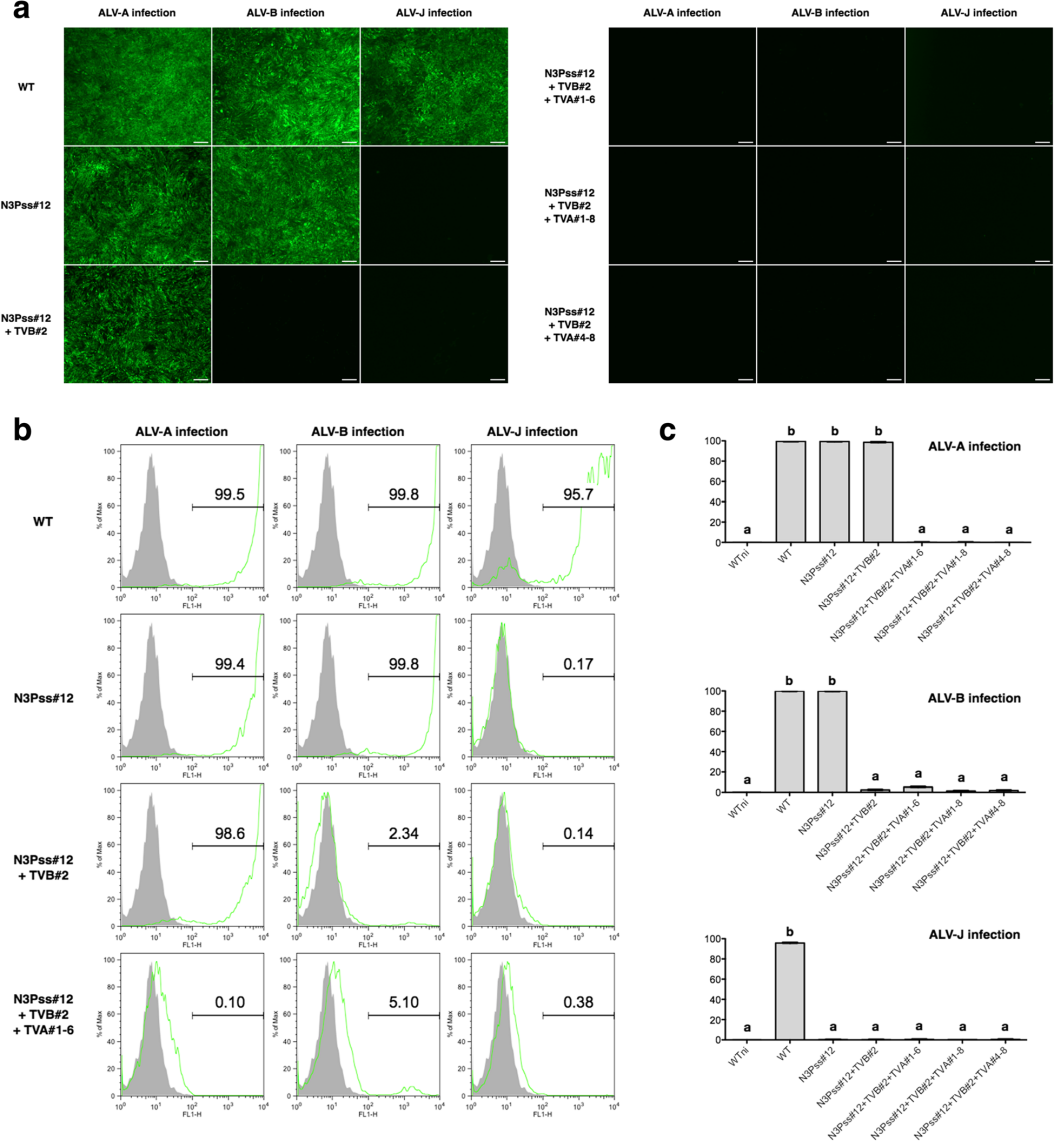
Infection of *chNHE1, tvb, and tva*-modified DF-1 clones with avian leukosis virus subgroup A, B, and J, followed by flow cytometry analysis. **a** Expression of GFP by virus-infected DF-1 clones. Five DF-1 clones were examined under a fluorescence microscope. Scale bar = 100 μm. **b**, **c** Flow cytometry analysis of virus-infected DF-1 clones. Gray peaks indicate the population of wild-type (WT) cells not infected with ALV subgroup A (negative control) and green peaks indicate the population of DF-1 clones infected by ALV subgroup A. The numbers on the histograms represent the mean of triplicate replications. WT DF-1 fibroblasts were used as the control (positive control). **c** Data represent the mean ± SEM of triplicate replications. Different letters (a, b) indicate significant differences (*P* < 0.05)

To identify the susceptibility of *chNHE1-*, *tvb-,* and *tva*-modified DF-1 clones to infection by ALV, we infected each clone with GFP-expressing marker viruses belonging to ALV subgroups A, B, and J, respectively. GFP expression was detected when the *chNHE1*-modified clone (N3Pss#12) was infected with either ALV subgroup A and B, but not upon infection with ALV subgroup J. In addition, GFP expression was detected only when *chNHE1*/*tvb*-modified clones (N3Pss#12 + TVB#2) were infected with ALV subgroup A, but not upon infection with ALV subgroups B and J. No GFP expression was detected in *chNHE1*/*tvb*/*tva*-modified DF-1 clones (N3Pss#12 + TVB#2 + TVA#1–6, N3Pss#12 + TVB#2 + TVA#1–8, and N3Pss#12 + TVB#2 + TVA#4–8) after infection by ALV subgroups A, B, and J (Fig. 6a). Subsequent flow cytometry analysis showed marked GFP expression by DF-1 clones harboring WT host genes (Fig. 6b and c).

## Discussion

Successfully controlling infectious diseases is one of the most important challenges facing the poultry industry. Enhancing economic traits using programmable genome editing technologies such as CRISPR/Cas9 has merit in terms of reducing the time required to generate a desired genotype in livestock species [23]. In particular, programmable genome editing technology can be applied directly to develop resistance to specific viral diseases such as ALV, for which the host receptors have been identified. Here, we used genome editing technology to develop cell lines that are resistance to infection by multiple ALVs; this step will precede development of genome-edited chicken lines.

As reported previously, ALV subgroup A enters host cells via the TVA receptor [7]. Deletion of introns from the *tva* gene disrupts mRNA splicing and/or induces frameshift mutations in exon 2, thereby altering host cell susceptibility to infection by ALV subgroup A [13–15]. Here, we induced a frameshift mutation into the *tva* gene rather than modifying the intron region to confer resistance to ALV subgroup A. The two CRISPR/Cas9 vector constructs used induced nucleic acid mutations within the targeted regions, and the pattern of mutations correspond to those observed in our previous studies [19, 20]. The results indicated that the CRISPR/Cas9-based gene editing system is an efficient tool for targeting specific regions of the chicken genome. However, we were unable to induce the exact mutation in the *tva* gene that is reported to confer resistance [13]. The experiments could be improved by using HDR-based approaches as well as by evaluating off-target mutations.

By contrast, the results of virus challenge experiments showed that nucleotide mutations in the *tva* gene that result in frameshift mutations are crucial for ALV subgroup A entry. The TVA receptor belongs to LDLR family; indeed, the LDLa plays an important role in receptor function [24]. Exon 2 of the *tva* gene contains the LDLa; therefore, we suggest that frameshift mutations causing disruption of the LDLa domain would lead to misfolding of the TVA protein. Furthermore, the results of virus challenge experiments in Asp67-deleted (TVA#1–5) and Asp67- and Glu68-deleted (TVA#1–2) DF-1 clones (these mutations do not cause a frameshift) also conferred resistance to ALV subgroup A. These results correspond with those published in previous studies examining ALV-A susceptibility linked to the TVA receptor [25]; two acidic amino acids in the carboxy-terminal portion of TVA receptor and the frameshift mutations are indispensable for infection by ALV subgroup A. Previous studies show that Trp52, Asp55, His57, Asp59, Asp65, and Glu66 of the quail TVA protein are calcium-coordinating sites for the TVA LDL-A module and are required for proper protein folding. Furthermore, His57 and Trp67 of the quail TVA protein act as viral contact residues and are involved directly in binding to EnvA [26, 27]. Protein structure analysis revealed that the LDLR region harboring these residues is highly conserved in different chicken TVA receptor isoforms. These results suggest that the in-frame *tva*-modified DF-1 clones have impaired function due to improper protein folding caused by a lack of calcium binding residues. Also, they do not bind to EnvA due to lack of viral contact residues. Moreover, we showed that overexpression of WT TVA restores susceptibility to infection by ALV subgroup A viruses. However, differences in susceptibility were noted between cell lines containing the WT TVA overexpression cassette, possibly due to the differences in *piggyBac* integration sites and copy number of transgene [22]. Meanwhile, modified TVA receptors could not restore susceptibility to infection by ALV subgroup A viruses in *tva*-modified DF-1 clone. Taken together, the results indicate that the TVA receptor is necessary for entry of ALV subgroup A viruses and could therefore be targeted to alter the immune characteristics of poultry flocks.

We also found that sequential disruption of ALV host receptors chNHE1, TVB, and TVA confers resistance to ALV subgroups A, B and J, respectively. We examined this by modifying the host receptor genes in chicken DF-1 fibroblasts by sequential introduction of CRISPR/Cas9 gene editing vectors. The results demonstrated that altering multiple genes can be achieved; this technique can then be used to develop multiple gene knock-out cell lines. Subsequent virus infection experiments showed that disruptions of the *tva-*, *tvb-,* and *chNHE1*-encoded receptors conferred simultaneous resistance to ALV subgroups A, B and J. As expected, the results also confirmed that ALV subgroups A, B and J do not share the same receptors for entry into cells, despite the close sequence similarity between ALV subgroups A and B [28]. By understanding interactions between ALVs and host receptors at the genetic level we may be able to predict likely host receptors for any emerging subgroup of ALV.

## Conclusion

We conducted multiplex genome editing of chicken DF-1 fibroblasts and showed that sequential disruption of host receptors for ALV subgroups A, B, and J confers resistance. To gain further insight into virus-host interactions, as well as into virus evolution, we will analyze the susceptibility of these cells to other virus subgroups (such as ALV subgroup C and the recently reported subgroup K). As a next step, we will apply the genome editing strategy to germline competent cells, including primordial germ cells or spermatogonial stem cells [29, 30], to generate novel multi-disease resistant chicken lines as an approach to controlling major avian diseases. Indeed, *NHE1*-null fibroblasts show impaired homeostasis, manifested by reduced adhesion, loss of polarity, and reduced motility and chemotaxis [31]. However, chicken cell lines naturally resistant to ALV subgroups A and B are available (e.g., inbred chicken line C and line 7_2_), as are avian species harboring mutations in the Trp38 of the NHE1 receptor [13, 18]. These results suggest that the ALV host receptors may be not be critical, or that other receptors compensate for a specific function of each receptor. To evaluate the function of each receptor, physiological and molecular analyses of immune responses and cell homeostasis in receptor gene-modified cells or birds are needed.

## Additional file

Additional file 1: **Figure S1.** Sequences of the targeted regions within *tva*-modified DF-1 clones. Red arrows indicate TVA#1 and TVA#4 targeting regions. A reference sequence is shown. **Figure S2.** Sequences of targeted regions within (a) *chNHE1*, (b) *chNHE1* and *tvb*, and (c) *chNHE1*, *tvb,* and *tva*-modified DF-1 clones. Red arrows indicate the (a) NHE1#3, (b) TVB#2, and (c) TVA#1 and TVA#4 targeting regions. Reference sequences are shown. (DOCX 848 kb)

## Abbreviations

ABAM: Antibiotic–antimycotic
ALV: Avian leukosis virus
ANOVA: Analysis of variance
chNHE1: Na^+^/H^+^ exchange 1
CRD: Cysteine-rich domain
CRISPR/Cas9: Clustered regularly interspaced short palindromic repeats (CRISPR)/CRISPR-associated protein 9
DMEM: Dulbecco’s minimum essential medium
FBS: Fetal bovine serum
GFP: Green fluorescent protein
LDLa: Low-density lipoprotein receptors (LDLR) class A domain
PGCs: Primordial germ cells
SAS: Statistical analysis system
SSCs: Spermatogonial stem cells
TNFR: Tumor necrosis factor receptor
Trp38: Tryptophan residue at position 38
WT: Wild type
wtTVA: Wild type TVA receptor
