# Supplementary material for: Sequential disruption of ALV host receptor genes reveals no sharing of receptors between ALV subgroups A, B, and J
Source: J Anim Sci Biotechnol. 2019 Apr 2;10:23. doi: 10.1186/s40104-019-0333-x (PMC6444617; doi:10.1186/s40104-019-0333-x)
Supplement: Supplementary file 1 — Figure S1. Sequences of the targeted regions within tva-modified DF-1 clones. Red arrows indicate TVA#1 and TVA#4 targeting regions. A reference sequence is shown. Figure S2. Sequences of targeted regions within (a) chNHE1, (b) chNHE1 and tvb, and (c) chNHE1, tvb, and tva-modified DF-1 clones. Red arrows indicate the (a) NHE1#3, (b) TVB#2, and (c) TVA#1 and TVA#4 targeting regions. Reference sequences are shown. (DOCX 848 kb) [file 40104_2019_333_MOESM1_ESM.docx]

**Additional file 1**

**
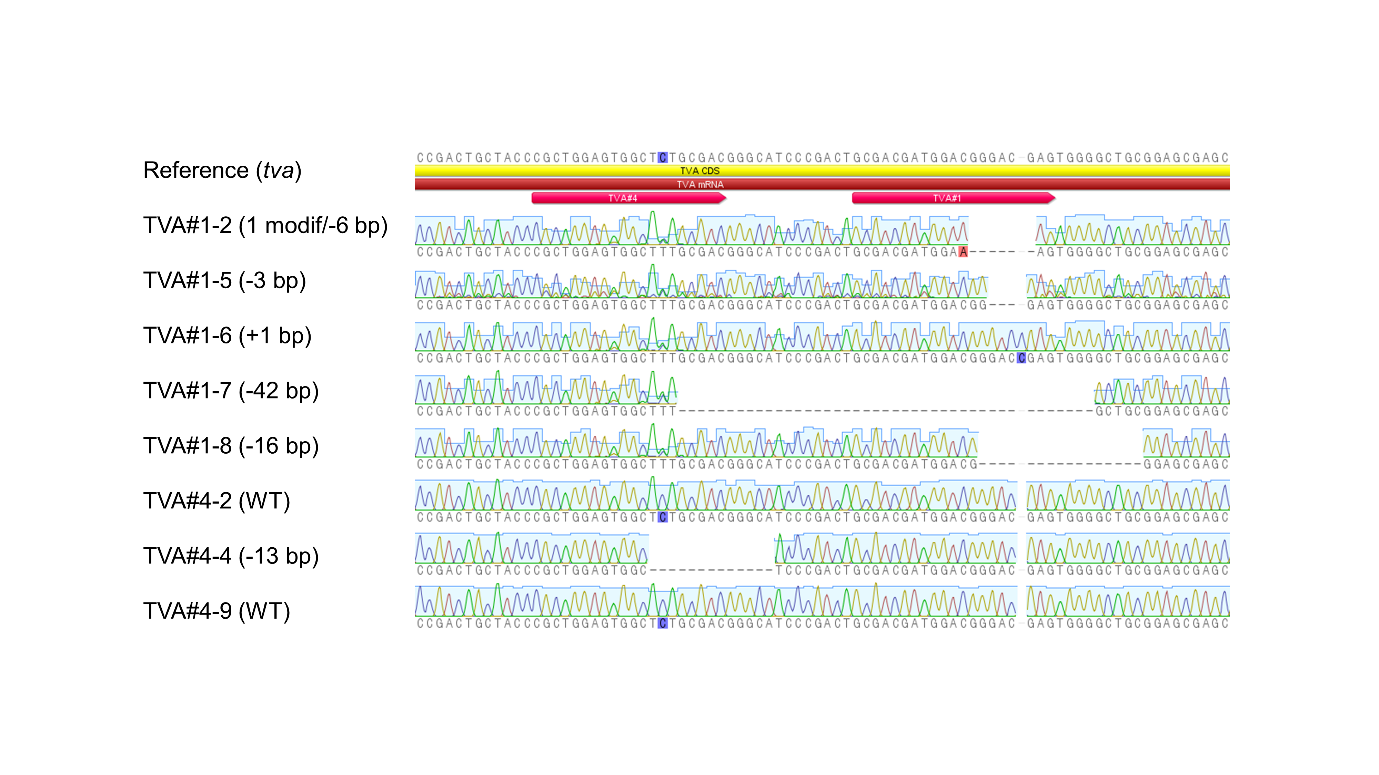
**

**Figure S1.** Sequences of the targeted regions within *tva*-modified DF-1 clones. Red arrows indicate TVA#1 and TVA#4 targeting regions. A reference sequence is shown.


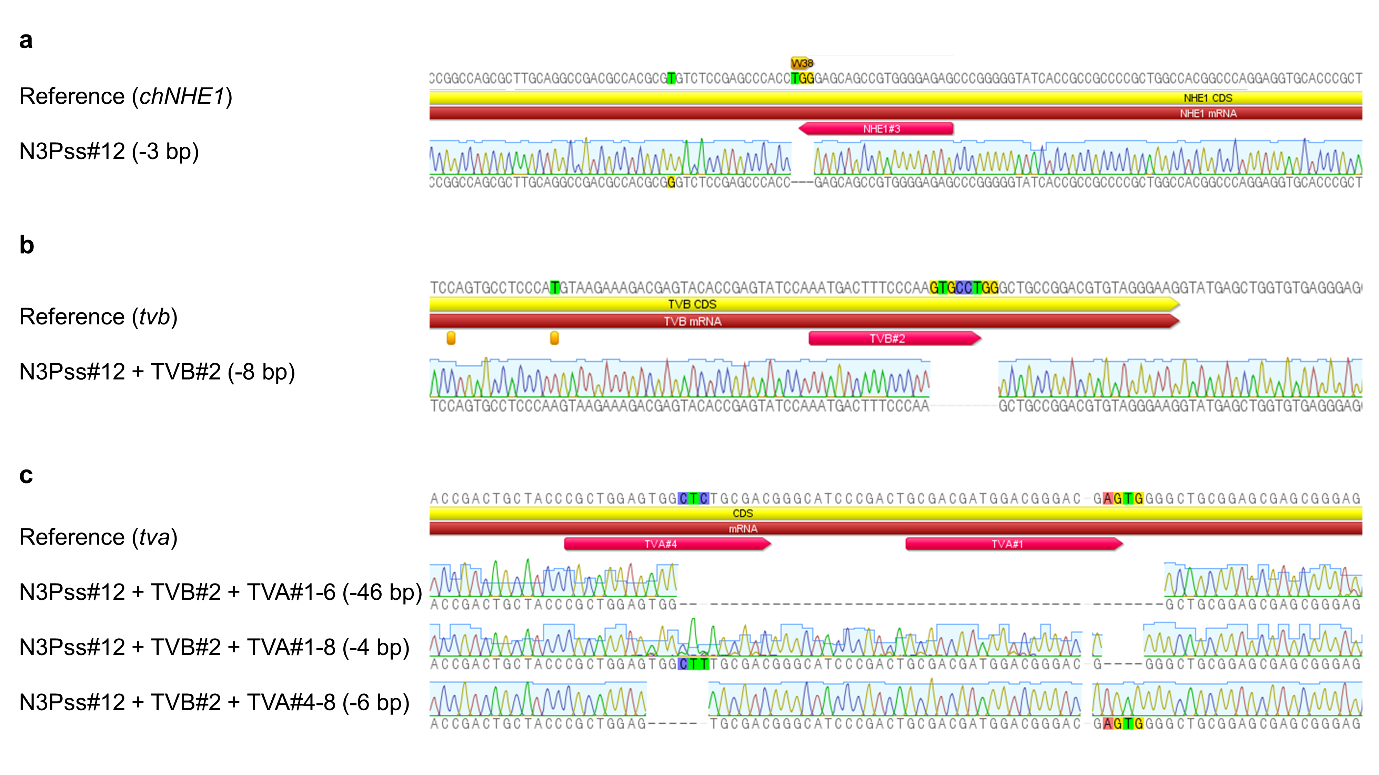


**Figure S2.** Sequences of targeted regions within (a) *chNHE1*, (b) *chNHE1* and *tvb*, and (c) *chNHE1*, *tvb,* and *tva*-modified DF-1 clones. Red arrows indicate the (a) NHE1#3, (b) TVB#2, and (c) TVA#1 and TVA#4 targeting regions. Reference sequences are shown.
